# Suitable Scales; Rethinking Scale for Innovative Integrated Care Governance

**DOI:** 10.5334/ijic.5468

**Published:** 2020-01-08

**Authors:** M. M. N. Minkman

**Affiliations:** 1 Tilburg University/TIAS, NL; 2 Vilans Center of Excellence in long term Care, NL; 3 International Foundation for Integrated Care, UK

**Keywords:** scale, integrated care governance, networks

## Abstract

For organising person entered care, an important issue is how to deal with scale. This addresses what to organise on what level (in the neighbourhood, local, in the region, or national). With the increasing complexity of organising integrated care in networks, scale issues are an ingredient of integrated care governance. However, there is a lack of empirical studies that treat scale as an object of study in itself. Scale is an outcome of the interplay between many different interests, values and perceptions of people involved in the broader social and political processes. Five factors for suitable scales are discussed, emphasising the relevance for integrated care governance. These factors show, that the classical micro-meso-macro thinking oversimplify reality and more knowledge about suitable scales is required.

## Introduction

In many countries integrated care is seen as the way forward. To professionals and laypeople, and also at a policy level, the urgency to better align and organise care and support around people in need is evident [1,2]. However, we know that there is no one-size-fits-all solution to achieve this. Context is important. One line of thinking, which should be better understood in my opinion, is the question of how to deal with scale. On the one hand there is the preference for organising support locally in the community, and on the other hand there is a need for more overarching networks and greater collaboration. In some countries we see shifts from centralised care systems towards more decentralised systems and also the reverse [3,4]. So the question remains – what kind of care fits what scale? What should be organised locally and what would work better on a larger scale? The classic ‘micro, meso and macro’ level classification seems no longer to meet our current challenges.

## Trends

Recent trends underline the need for policy-makers and healthcare leaders to rethink scale. One development is the increasing focus on person-centredness, which is reflected in how we organise care, how we value the importance of including the family in care decisions and in the development of personalised tailored medicine. The health care *facility* itself, such as a hospital or a primary health care facility, becomes less important whereas the location of care and support is more often where the person is; which is at home. This is often also referred to as de-institutionalisation [5]. In alignment with person-centredness, there is an awareness that healthy societies are driven by community embedded population health approaches. Creating vital communities, in which there is an awareness and urgency for healthy but also for happy living. Secondly, we see that to meet the complex needs of citizens (which we do not call patients), services may be required that fall outside the domain of traditional health care. Creating valuable connections with other sectors like education, housing, work or leisure is the real challenge in this case [6]. Furthermore, many countries, due to demographics and migration patterns, face a shortage of health care staff. This creates an urgent need for new and breakthrough models of care and requires investment in caring communities. These communities as such create new dilemma’s. For instance, how much load can ‘vital elderly’ (under 80) actually carry in their increasing task as informal carer? Not only is staff a scarce resource, the rising health and social care costs are of growing concern. For example, in (my country) the Netherlands the future costs for long term care are a real issue [7]. A final development which I would like to highlight, is the change in how we perceive ‘care at a distance’. With digital technology, a teleconsultation in Australia (from a European point of view) is no longer regarded as ‘far away’ and experts from all over the world can be consulted for a second opinion.

## The need for rethinking scale

These developments have an enormous impact on how we organise our health and social care. Whereas the healthcare organisation as an entity was for decades the primary focus for optimalisation, creating valuable connections in network-like organisational models including interdisciplinary teams is the new paradigm. This has consequences for how we define and execute responsibilities and determine who is accountable for what. Leadership, accountability and supervision have different characteristics in integrated care settings than in traditional more hierarchical organisational models. They are important elements in integrated care governance [8,9]. For leaders and policy-makers who have to make integrated care governance happen, an important question in integrated care governance is how to deal with scale. The urgency to organise care in another modus, to deal with new and other scales of services and collaborations, points out the unsustainability of ‘classic’ organisational governance. Knowledge about suitable scales is therefore important in our search for delivering the best value for people [10]. Surprisinlgly, scale has not often been a subject of research yet.

How can we look at scale? Traditionally scale is an important subject in geography. Robertson [11] describes scales as *“graduated series, usually a nested hierarchy of bundled spaces of different sizes”*. Scales are often described in terms of a continuum, layers or a hierarchy – for instance micro, meso and macro; large, medium and small; and local, regional, national and global. Although this may imply that scales have a neat vertical structure, Taylor and Spicer [12] emphasize, in line with insights from human geography, that there is no inherent and absolute hierarchical relationship between scales. Sometimes ‘lower’ levels are much more important than ‘higher’ levels, as for example in Nordic countries like Sweden where the municipality has a bigger say in local health and social care policy than the national government level [4]. In Finland it is interesting to note that although decentralisation has been implemented, current policies actually focus on partial re-centralization into 18 new counties instead of the municipalities [3]. Postma concludes that it is essential to understand how different scales relate to each other and how they may become more or less important over time. This can have a significant impact on strategy and national policies [5]. For purposes in integrated care settings, it is from this point of view important to realise that increasing scale is not the same as acting on a ‘a larger or on a national scale’.

## Scale as a social and political construct

Postma [5], who has researched scale issues in health care extensively, argues that scale is often too easily taken for granted. Studies show that scale is not a neutral set of pre-given levels at which social processes take place. Instead it is a subjective, contingent way of seeing and organizing [13,14,15]. In the Netherlands this is reflected in what I sometimes call ‘scale confusion’. In the same geographical area, the same health and social care providers started to develop integrated care networks for people with dementia, palliative care needs and the vulnerable elderly. The degree of overlap, both in target groups, strategic partners and geographic area led to new questions being asked about how to increase efficiency in all of these collaborations [15]. This illustrates that the existence of scale is the result of human (inter)actions. It is not determined by the inherent nature of things; it is not inevitable but subjective [16]. Postma [5] underlines the concept of seeing scale as a social and political construct: the definition of scale, the scale at which healthcare is provided, and how this is achieved, is an outcome of the interplay between many different interests i.e. the values and perceptions of people involved and the broader social and political processes. Healthcare professionals and organisations in integrated care are intertwined in multiple networks and collaborations at different scales. This means that there is no such thing as one scale or one optimal scale. The social constructivist perspective, acknowledges that there are always multiple scales to consider, multiple values that play a role and diverse perceptions of people [5].

## Scale and integrated care governance

Rethinking what is a suitable scale relates directly to the importance of integrated care governance. Our challenges in societies are so big and need interdisciplinary approaches and solutions, that single provider- or organisation strategies often do not fulfil the needs of citizens. We do not only see this in health care, but also for instance environmental and climate issues or energy transitions are only solvable by interdisciplinary and domain overarching aligned solutions. In health and social care however, governance of organisations defined as the total package of leadership, accountability and supervision, do follow the traditional approaches focused on the steering and control of the organisation [8]. This governance no longer fits working on societal aims of these complex issues. For example, an inventory in 135 health- and social care networks in the Netherlands which have the aim to serve clients better, stated that in one-third of them the interests of clients are not taken into account in their decisions as a network. If clients are involved, this is often via the professionals. The networks illustrate that they struggle how to organise their governance and on what scale (population, target group, geographical area) [17]. Traditional governance *within* organisations does not match the needed governance *between* organisations. Models based on network governance that are more horizontal, non-hierarchic, based on trust as a basic value seem to be more fitting. Working with those models however, asks for a complete re-thinking of the roles and internal processes of boards and supervisors. Also, it asks for re-thinking what governance mechanisms there are for decision making, when there is no formal hierarchy between partners, but dependencies do exist [29]. Integrated care governance reshapes also traditional accountability mechanism. Accountability goes beyond being responsible towards ‘who can pay or who can punish’ (health insurers or inspectorates) but also towards the society or (local) citizens, especially when they are increasingly becoming partners and co-producers of (informal) care [18]. When the role of citizens themselves changes, community governance including democratic representation, intertwined with health- and social care networks on a suitable scale is the new landscape [19].

## Values and scale

The relationship between scale and values is an interesting one. Studies aiming to define the optimal scale are traditionally designed from an economic standpoint. For instance, if a decision needs to be taken about merging or expansion, an optimal scale may be determined based on economic outcomes. Other studies in the public administration and organisational literature have a broader scope for example by researching the relationship between the size of an organisation and the quality of the products and services. The work of Zonneveld [20,21] about the normative aspects of integrated care, shows that integrated care, because of its inherent multidimensionality, is driven by multiple values. These values cover a wide range from ‘holistic’ and ‘reciprocal’ to ‘goal-oriented’ and more. Ongoing studies show that the perspective of persons involved in integrated care (professionals, management etc) has consequences for the values considered to be important. But even studies that take multiple values into account when searching for the optimal scale are problematic. Since values can be intrinsically conflicting, so do the definitions of optimal scale based on these values [22,23]. Lastly, there is also a subjective component to scale. Whereas the popular concept of ‘Buurtzorg’ (a homecare organization) in the Netherlands, which works with very local nurse-led teams, is experienced by clients as ‘small scale’, the organisation actually employs over 10,000 nurses and operates in 24 countries [24].

## Factors for suitable scales

Identifying the most suitable scale is complex or–as Postma argues–impossible. However, for integrated care governance and policy-makers, scale is an important issue that has to be dealt with. From an overview of the literature, I would like to suggest five factors which should be taken into consideration when dealing with scale issues.

First, there is a relationship between scale and volume. Either a high number of people with similar needs or a group of people who are willing to support each others’ diverse needs, may yield options in terms of local scales. For instance, groups of elderly who would like to live at home as long as possible in old age communities, or caring communities where people at different stages of life can support each other. Using the potential of local communities is still in it’s early stages of development [25]. Integrating services and care really on a widespread community level and seeing citizens as co-producers of care and support, would really increase a widespread scale.

A second factor regarding scale is the need for specialised knowledge. Research shows that there is a relationship between quality and outcomes of services and volume. Treatments that require specialised knowledge benefit from a high volume of this specific treatment that is organised in a concentrated way. More focus and consolidation in knowledge of specialised work, translates into better outcomes [26,27,28]. This is the reason that discussions about the type of medical care to be delivered by particular hospitals (general hospitals, academic hospitals) are also essential for reducing costs. Specialised knowledge needed for small groups of people who live spread out over a large area, could lead towards a concentration of knowledge on a small scale. However, this is not necessarily concentration in terms of a physical location; a digital connection, telehealth and specialised large scale working teams can also serve as organisational models.

A third factor is the scale of the ‘reasonable responsible entity (RRE)’. By this I mean the logic behind who or what is a workable size or model that can oversee the service and take responsibility for it. This could be the municipality or the county or something else. In large scale organisations, it is not possible for the board to oversee everything although they take final responsibility. It is actually un necessary, because others in the organisation are tasked with the supervision and responsibility for specific parts of the whole. However, there are also examples which show that if the size increases and organisational structures are unclear, there is a risk of losing control. For management and staff, they value belonging to a certain entity. Carers in a nursing home often feel more connected to the location where they work, than to the larger holding which employs them. In integrated care there is the extra dimension of overseeing individual contributions to the whole, while also having a shared responsibility for the whole. In integrated care and services, there are almost always multiple organisations and stakeholders involved. In integrated care governance, taking responsibility together for the whole while there are unequal contributions, is therefor a challenging feature. Having a responsibility for the own organisation and at the same time participating in multiple networks can give tensions between the values of the societal aims and those of the organisation. The more partners involved in the RRE, the bigger the coordination and governance challenge. A suitable scale fits the RRE, which can differ over time, differ over geographical areas (multiple municipalities together for instance or a single one) and also differ within a country.

Fourthly, there is a ‘local reasoning’ in the case of existing networks and collaborations based on historical developments and current boundaries. In the Netherlands networks in health and social care are numerous and diverse. Borders are sometimes geographic, but they may also be provider-related or based on health insurance-related definitions. Although ‘local reasoning’ need to be to be taken into account, they should also be reviewed to establish whether they are still applicable or require more flexibility to current challenges. ‘Local reasoning’ encompasses human interactions and relationships which are generally based on trust. Rethinking scale never starts from scratch. Including characteristics and history of specific contexts, and also democratic representation of involved stakeholders or citizens encompasses the need for local reasoning.

Lastly, the fifth factor when considering appropriate scale concerns underlying values. The values which are important to different stakeholders, professionals, laypersons or policymakers is reflected in their policies and decision making. We know that decision making in integrated care networks is complex. It has to balance inclusiveness (involving all related stakeholders) versus efficiency (coming to decisions in an acceptable timeframe) [26]. An awareness about what values are key when making crucial decisions about complex scale issues could be supportive.

## Conclusions: beyond the ‘micro-meso-macro’ thinking

Overall, there is a need for more knowledge about suitable scale. This is relevant for both policy makers, healthcare leaders, professionals and laypersons. ‘Scale working’ and being able to adapt to multiple scales could become a key competence in the future. The multiplicity of scale is a fundamental issue for integrated care and integrated care governance. Conceptual hierarchies like ‘micro, meso and macro’ as used in the Rainbow model [30] oversimplify reality and do not sufficiently support integrated care development in practice. Rethinking and researching scale issues is therefore necessary. There is a lack of empirical studies that treat scale as an object of study in itself [5]. Studies of innovative organisational models and re-structuring examples are available, however they generally do not explore scale issues and their context. This would require research designs to go beyond the traditional evaluations, which usually do not investigate context, scale dynamics and adaptiveness sufficiently [31]. Research methods that both capture these dynamics and connect practice and science by mutual learning, are necessary to support our quest for suitable and sensible scales.
